# Investigation of Predictors to Achieve Acceptable Lung Dose in T-Shaped Upper and Middle Esophageal Cancer With IMRT and VMAT

**DOI:** 10.3389/fonc.2021.735062

**Published:** 2021-10-07

**Authors:** Yan Shao, Hua Chen, Hao Wang, Yanhua Duan, Aihui Feng, Ying Huang, Hengle Gu, Qing Kong, Zhiyong Xu

**Affiliations:** ^1^ Department of Radiation Oncology, Shanghai Chest Hospital, Shanghai Jiao Tong University, Shanghai, China; ^2^ Institute of Modern Physics, Fudan University, Shanghai, China

**Keywords:** esophageal cancer, intensity-modulated radiotherapy (IMRT), volumetric-modulated arc radiotherapy (VMAT), total lung dose predictor, cutoff point

## Abstract

**Purpose:**

The purpose of this study is to investigate whether there are predictors and cutoff points that can predict the acceptable lung dose using intensity-modulated radiation therapy (IMRT) and volume-modulated arc therapy (VMAT) in radiotherapy for upper ang middle esophageal cancer.

**Material and Methods:**

Eighty-two patients with T-shaped upper-middle esophageal cancer (UMEC) were enrolled in this retrospective study. Jaw-tracking IMRT plan (JT-IMRT), full-arc VMAT plan (F-VMAT), and pactial-arc VMAT plan (P-VMAT) were generated for each patient. Dosimetric parameters such as MLD and V20 of total lung were compared among the three plannings. Ten factors such as PCTV_inferior_ length and PCTV_inferior_ length/total lung length were calculated to find the predictors and cutoff points of the predictors. All patients were divided into two groups according to the cutoff points, and the dosimetric differences between the two groups of the three plans were compared. ANOVA, receiver operating characteristic (ROC) analysis, and Mann–Whitney U-test were performed for comparisons between datasets. A p <0.05 was considered statistically significant.

**Result:**

The quality of the targets of the three plannings was comparable. The total lung dose in P-VMAT was significantly lower than that in JT IMRT and F-VMAT. Monitor unit (MU) of F-VMAT and P-VMAT was significantly lower than that of JT IMRT. ROC analysis showed that among JT IMRT, F-VMAT, and P-VMAT, PCTV_i-L_, and PCTV_i-L_/TL_L_ had diagnostic power to predict the suitability of RT plans according to lung dose constraints of our department. For JT IMRT, the cutoff points of PCTV_i-L_ and PCTV_i-L_/TL_L_ were 16.6 and 0.59. For F-VMAT, the cutoff points of PCTV_i-L_ and PCTV_i-L_/TL_L_ were 16.75 and 0.62. For P-VMAT, the cutoff points of PCTV_i-L_ and PCTV_i-L_/TL_L_ were 15.15 and 0.59. After Mann–Whitney U-test analysis, it was found that among the three plannings, the group with lower PCTV_i-L_ and PCTV_i-L_/TL_L_ could significantly reduce the dose of total lung and heart (p <0.05).

**Conclusion:**

PCTV_i-L <_16.6 and PCTV_i-L_/TL_L_ <0.59 for JT IMRT, PCTV_i-L <_16.75 and PCTV_i-L_/TL_L_ <0.62 for F-VMAT and PCTV_i-L <_15.15, and PCTV_i-L_/TL_L_ <0.59 for P-VMAT can predict whether patients with T-shaped UMEC can meet the lung dose limits of our department.

## Introduction

Esophageal cancer (EC) is one of the most common malignant gastrointestinal tumors in the world (1) and also the sixth leading cause of cancer-related deaths (2). In China, esophageal cancer incidence and mortality rate account for more than half of the world (3). At present, surgery is still the preferred method for patients with EC. However, for patients with locally advanced or distant metastases who are inoperable or unwilling to operate, radical chemoradiotherapy is considered standard treatment (4).

The esophagus is in a unique location. It is close to the heart and surrounded by the lungs. Therefore, one of the challenges of radiotherapy planning for EC is to accurately deliver the radiation dose and minimize cardiopulmonary toxicity (5). For upper and middle esophageal cancer (UMEC), considering the risk of mediastinal and cervical lymph nodes metastasis, the radiotherapy target is usually large. It looks like a T in the anteroposterior direction, so we call it T-shaped. In the design of a radiotherapy plan, more attention should be paid to the dose delivered to normal tissues. In order to reduce the toxicity of normal tissues and improve the therapeutic effect, radiotherapy technology has been continuously developed (6, 7). Intensity-modulated radiation therapy (IMRT) can improve tumor coverage while reducing the dose to surrounding normal tissues (8–11). Studies have shown (12) that when comparing the long-term prognosis of esophageal cancer after 3D-CRT and IMRT radiotherapy, the IMRT group has longer overall survival (OS) and a lower local recurrence rate. IMRT includes jaw tracking IMRT and fixed-jaw IMRT. Volume-modulated arc therapy (VMAT) is a new type of IMRT method. By dynamically modulating the angular dose rate and the movement of the multileaf collimator, a highly conformal dose distribution can be obtained while shortening the treatment time (13). VMAT includes full arc VMAT and partial arc VMAT. The current technologies based on linear accelerators for the treatment of esophageal cancer are mainly IMRT and VMAT.

Esophageal lacks serosal layer, and lymphatic spread is common. Therefore, radiotherapy for EC usually has a large longitudinal safety margin, leading to a high dose delivered to the lungs. Radiation pneumonitis (RP) is a common complications of chest radiotherapy (14, 15). RP can induce emphysema, pulmonary fibrosis, and other problems, which affect the life quality of patients and even endanger the life of patients in severe cases (16). Studies have shown that RP incidence mainly depends on the dose and volume of the irradiated lungs (17). Therefore, the radiation of the lungs should be minimized in radiotherapy for EC. Some researchers have studied the dosimetric comparison of different radiotherapy techniques for patients with EC (18–21). Kataria et al. (18) compared VMAT and IMRT techniques in middle EC and found that VMAT reduced the doses to lungs and heart under the same dose distribution of target. Chen et al. (20) compared the dosimetric effects of jaw tracking partial arc VMAT, full arc VMAT, and IMRT technology in the treatment of upper esophageal cancer. It was found that compared with IMRT, full arc VMAT significantly increased the V5 of lungs, but partial arc VMAT did not. Based on the above researches, we envisioned whether one or more predictors can be found by expanding the number of enrolled patients. The predictors could predict whether the plan can meet the constraints of lungs before designing the plan. Bolukbas et al. (22) found that lung volume and lung volume/PTV volume are acceptable predictors of lung dose using TOMO by contouring the virtual radiotherapy volume of upper EC. It provides a method for the design of treatment plans based on the linear accelerator for T-shaped UMEC. On this basis, we added eight more factors to find genuinely effective predictors. Unlike Bolukbas et al. (22), our study is based on real targets of patients, and the predictors and cutoff points identified are closer to the real clinic situation.

In this study, we included actual patients with T-shaped EC. All patients were treated with linear accelerators. Jaw tracking IMRT plan (JT IMRT), full arc VMAT plan (F-VMAT), and partial arc VMAT plan (P-VMAT) were generated for each patient. Ten factors including lung volume and lung volume/PTV volume were calculated to evaluate the effects to the lungs dose in different plans. The predictors were determined, and the cutoff points in different plans were found. We hope that the results can improve the efficiency of plan design and assist dosimeters in choosing a more appropriate treatment methods for patients with T-shaped EC treated by linear accelerators. This may provide a direction for individual treatment of patients.

## Material and Methods

### Patients Characteristic

From July 2017 to December 2018, 82 patients with T-shaped esophagus cancer were included in this retrospective study. All patients were placed in supine position, fixed with a thermoplastic mask, and their arms placed on both sides of body. MX4000 CT Scanner System (Philips Medical Systems) was used for CT scanning. The scanning range was from the upper edge of the second cervical spine (the base of the skull) to the lower edge of the second lumbar spine, with a thickness of 5 mm. The scanned images were transferred to Philips Pinnacle 9.10 treatment planning system (Philips Healthy, Fitchburg, WI, USA) *via* the network.

### Delineation of Target and Organs at Risk

Target and organs at risk (OARs) were contoured by experienced radiation oncologist. Gross tumor volume (GTV) were delineated on CT images regarding to esophagography, esophagoscopy images, and pathology reports. Considering the setup error, respiratory movement, and other errors, GTV was isotropically expanded by 6 mm to form the planning gross tumor volume (PGTV). Clinical target volume (CTV) was defined as GTV plus bilateral supraclavicular lymph nodes and superior mediastinal lymph nodes. CTV isotropically expanded 0.6–1.0 cm to form planning clinical target volume (PCTV). OAR included spinal cord, total lung, and heart. Total lung was defined as right lung plus left lung minus GTV. In order to facilitate the statistical results, apex of the total lung was used as the dividing line; the PCTV above the dividing line was named PCTV_superior_, and the PCTV below the dividing line was named PCTV_inferior_.

### Treatment Planning

Three simultaneous integrated boost plans were generated for each patient: JT IMRT, F-VMAT, and P-VMAT. All plans were designed by a senior physicist in the auto-planning module of the Pinnacle 9.10 planning system. Varian Edge linear accelerator (Varian, Palo Alto, CA, USA) with 6 MV photon beam was adapted. Direct machine parameter optimization (DMPO) algorithm was used, and heterogeneity of the tissue was considered. The grid resolution of the dose calculation was set to 3 mm. The prescribed dose of PGTV was 60.2 Gy/28 fractions, and the single fraction dose was 2.15 Gy. The prescription of PCTV was 50.4 Gy/28 fractions, and the single fraction dose was 1.8 Gy. All plans were normalized so that 100% of PGTV was covered by 95% of the prescribed dose.

For the JT IMRT plan, the beam angles were set to 210°, 300°, 330°, 0°, 30°, 60°, and 150° based on our clinical experience. Jaw motion was allowed in the planning system. For the F-VMAT plan, the beam angles were 180.1°–180° (CW, CCW). The beam angles for the P-VMAT plan were 180.1°–210° (CW, CCW), 300°–60° (CW, CCW), and 150°–180° (CW, CCW) (17). The beam angle could be adjusted slightly based on the actual situation of the patient.

According to the protocol of our department, total lung V5 ≤50%, V20 ≤25%, mean lung dose (MLD) ≤15 Gy, spinal cord ≤50 Gy, heart V30 ≤40%, heart V40 ≤30%, and mean heart dose (MHD) ≤26 Gy. A plan with all parameters within the dose constraints was considered as a qualified plan. Plans with at least one of the parameters above the dose constraints were considered as unqualified plans.

### Treatment Planning Evaluation

In this article, dosimetric differences of the three kinds of plans were compared, and dose–volume histograms (DVHs) were used to evaluate the dose of the target, total lung, heart, and spinal cord. Conformity index (CI), heterogeneity index (HI), D2, and D98 were used to evaluate the target. CI (23) formula is CI = V_T,ref_/V_T_ × V_T,ref_/V_ref_, where V_T,ref_ is the volume of PTV covered by prescription dose, V_T_ is the volume of PTV, and V_ref_ is the volume covered by prescription dose. The closer the CI is to 1, the better the conformability is. The formula of HI (20) is HI = (D2–D98)/Dp, where Dp is the prescription dose. The smaller the HI is, the better the uniformity is. The evaluation parameters of OARs involved V5, V10, V13, V20, V30, V40, and MLD of total lung and spinal cord Dmax, and V30, V40, and MHD of the heart. In addition, we also assessed the differences in monitor unit (MU) between different plans. γ was evaluated under the analysis standard of 3 mm/3% (10% low-dose threshold) with the Varian onboard measuring device portal dosimetry (PD, Varian Medical Systems); then, Eclipse system (Varian Medical Systems, Palo Alto, CA, USA) was used to compare the predicted dose and measured dose.

In order to find more comprehensive and accurate predictors of lung dose, as many parameters as possible were included in this study. Based on the published literature (22, 24) and years of clinical experience of our department, 10 parameters were finally counted in this study, namely, PCTV width (PCTV_W_), PCTV length (PCTV_L_), PCTV volume (PCTV_V_), total lung volume (TL_V_), total lung length (TL_L_), PCTV volume/total lung volume (PCTV_V_/TL_V_), PCTV_inferior_ volume (PCTV_i-V_), PCTV_inferior_ length (PCTV_i-L_), PCTV_inferior_ volume/total lung volume (PCTV_i-V_/TL_V_), and PCTV_inferior_ length/total lung length (PCTV_i-L_/TL_L_). These parameters consider factors such as target shape and target length and to a certain extent can comprehensively analyze the predictive ability of target parameters for lung dose. Through data processing, predictors and their cutoff points were found out. For each predictor, 82 patients were divided into two groups at the cutoff point of the predictor, and the dosimetric differences between the two groups of the three plans were compared.

### Statistical Analysis

SPSS 20.0 (IBM Corporation, Armonk, NY, USA) statistical software was used for analysis. In order to determine the statistical significance between the groups, ANOVA analysis was used. Receiver operator characteristic (ROC) curve was used to analyze whether factors such as PCTV_i-L_ and PCTV_i-L_/TL_L_ can predict the lung dose of the three radiotherapy plans. ROC curve analysis was used to analyze and evaluate the effect of binary classification. The independent variables are generally continuous variables. In this study, they referred to 10 factors such as PCTV_i-L_ and PCTV_i-L_/TL_L_. The dependent variables are generally binary variables. In this study, they referred to plan qualified and plan unqualified. Sensitivity referred to the ratio of the number of judged unqualified plans to the number of truly unqualified plans. Specificity referred to the ratio of the number of judged qualified plans to the number of truly qualified plans. Misjudgment rate referred to the ratio of the number of judged unqualified plans to the number of truly qualified plans, and its value is equal to 1 − specificity. Positive predicted value referred to the ratio of the number of truly unqualified plans to the number of judged unqualified plans. Negative predictive value referred to the ratio of the number of truly qualified plans to the number of judged qualified plans. Accuracy referred to the probability of correct judgment in all cases. ROC analysis was used to obtain multiple pairs of sensitivity and misjudgment rate (1 − specificity) by moving the point. The sensitivity was taken as the vertical axis and the misjudgment rate as the horizontal axis. Each point was connected to draw the curve, and then, the area under the curve was calculated, that is, AUC. The larger the AUC, the higher the judgment value. This method to find the cutoff point on the ROC curve was used to maximize the sensitivity and minimize the misjudgment rate. Jordan index was defined as the sum of sensitivity and misjudgment rate. Therefore, when the Jordan index reaches the maximum, the corresponding value is the cutoff point we are looking for. The sensitivity, specificity, and positive and negative predictive values were calculated when significant cutoff points were observed. Mann-Whitney U-test was used to compare groups with the non-normally distribution. A p < 0.05 was considered statistically significant.

## Results

A total of 82 patients’ data were collected in this study. The median age of the patients was 64 years (range, 46–85 years), including 68 men and 14 women. Detailed patients’ characteristics are shown in **Table 1**.

**Table 1:** Patient characteristics.

Characteristics	Number of Case
Age (years)	
Median	64
Range	46–85
Gender	
Male	68
Female	14
PCTV width (cm)	
Median	14.16
Range	10.6–22.3
PCTV length (cm)	
Mean	15.91
Range	12.4–21
PCTV volume (cc)	
Mean	529.82
Range	245.12–977.15
Total lung volume (cc)	
Mean	3,238.60
Range	1,704.50–5,926.76
Total lung length (cm)	
Mean	20.09
Range	15.5–25.5
PCTV volume/total lung volume	
Mean	0.17
Range	0.08–0.33
PCTV_inferior_ volume (cc)	
Mean	365.32
Range	152.96–582.79
PCTV_inferior_ length (cm)	
Mean	11.85
Range	7–18.8
PCTV_inferior_ volume/total lung volume	
Mean	0.12
Range	0.05–0.21
PCTV_inferior_ length/total lung length	
Mean	0.59
Range	0.39–0.94

**Figure 1:**
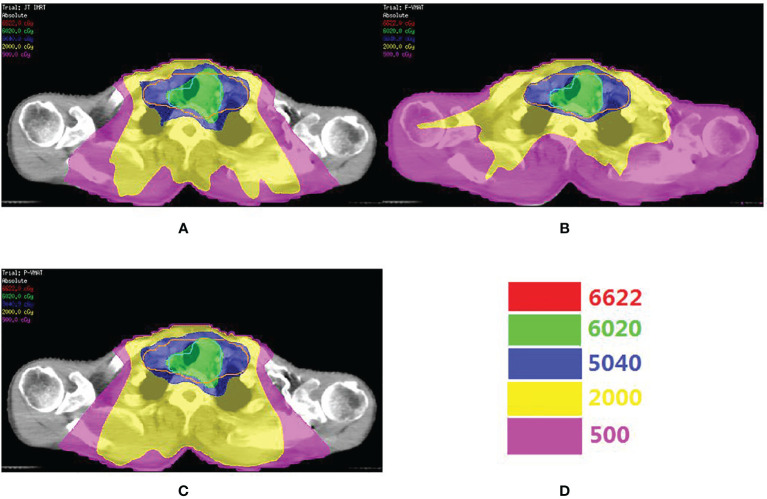
The comparison o dose distribution in JT IMRT **(A)**, F-VMAT **(B)** and P-VMAT **(C)** for one patient. The green outline is PGTV. The orange outlineis PCTV. **(D)** Shows the isodose line.

A total of 246 treatment plans were designed for 82 patients in this study. **Figure 1** shows the dose distribution of a patient with three delivery techniques. The comparison of the dosimetric parameters of PTV and OARs for the three plans is shown in **Table 2**. For the target, there was no statistical difference in all parameters between the three plans. For total lung, V5, V10, V13, V15, V20, and MLD in P-VMAT were lower than those in JT IMRT and F-VMAT, and there were statistical differences. There was no statistical difference in MHD between the three plans. The V30 and V40 of the heart in F-VMAT were lower than those in JT IMRT and P-VMAT, and there were statistical differences. The MU of F-VMAT and P-VMAT was significantly lower than that of JT IMRT. There was no statistical difference in γ passing rate between the three plans.

**Table 2:** Comparisons of dosimetric parameters of PTV and OAR.

	JT IMRT	F-VMAT	P-VMAT	p-value
PGTV				
D_2_ (Gy)	64.35 ± 0.78	64.30 ± 0.80	64.35 ± 0.79	0.91
D_98_ (Gy)	59.44 ± 0.72	59.63 ± 0.64	59.48 ± 0.79	0.21
CI	0.84 ± 0.04	0.85 ± 0.04	0.85 ± 0.03	0.16
HI	0.09 ± 0.02	0.08 ± 0.02	0.08 ± 0.02	0.53
PCTV				
D_2_ (Gy)	63.78 ± 0.99	63.67 ± 0.92	63.85 ± 0.96	0.49
D_98_ (Gy)	50.63 ± 1.02	50.38 ± 1.54	50.67 ± 2.51	0.09
Total lung				
MLD (Gy)	9.45 ± 1.91	9.51 ± 2.11	8.76 ± 1.69	0.02
V_5_ (%)	42.37 ± 10.50	47.21 ± 14.87	38.37 ± 10.62	<0.001
V_10_ (%)	30.86 ± 8.36	33.80 ± 10.80	27.29 ± 6.77	<0.001
V_13_ (%)	27.10 ± 7.00	27.76 ± 7.36	23.48 ± 5.23	<0.001
V_15_ (%)	24.57 ± 6.23	24.28 ± 5.59	21.22 ± 6.23	<0.001
V_20_ (%)	18.42 ± 3.80	17.04 ± 3.27	16.29 ± 3.02	<0.001
V_30_ (%)	8.92 ± 2.15	7.37 ± 1.92	8.83 ± 2.10	<0.001
Spinal Cord				
D_max_ (Gy)	44.06 ± 2.50	41.82 ± 3.76	43.92 ± 2.35	<0.001
Heart				
MHD (Gy)	6.62 ± 5.45	6.03 ± 4.35	6.90 ± 5.40	0.54
V_30_ (%)	8.47 ± 8.61	5.71 ± 4.99	8.72 ± 8.20	0.02
V_40_ (%)	4.12 ± 4.18	2.82 ± 2.60	4.54 ± 4.41	0.01
MU	795.93 ± 209.62	593.87 ± 106.16	544.60 ± 69.78	<0.001
γ (%)	99.95 ± 0.09	99.98 ± 0.05	99.95 ± 0.09	0.06

Highlighted text means γ passing rate.

**Figure 2:**
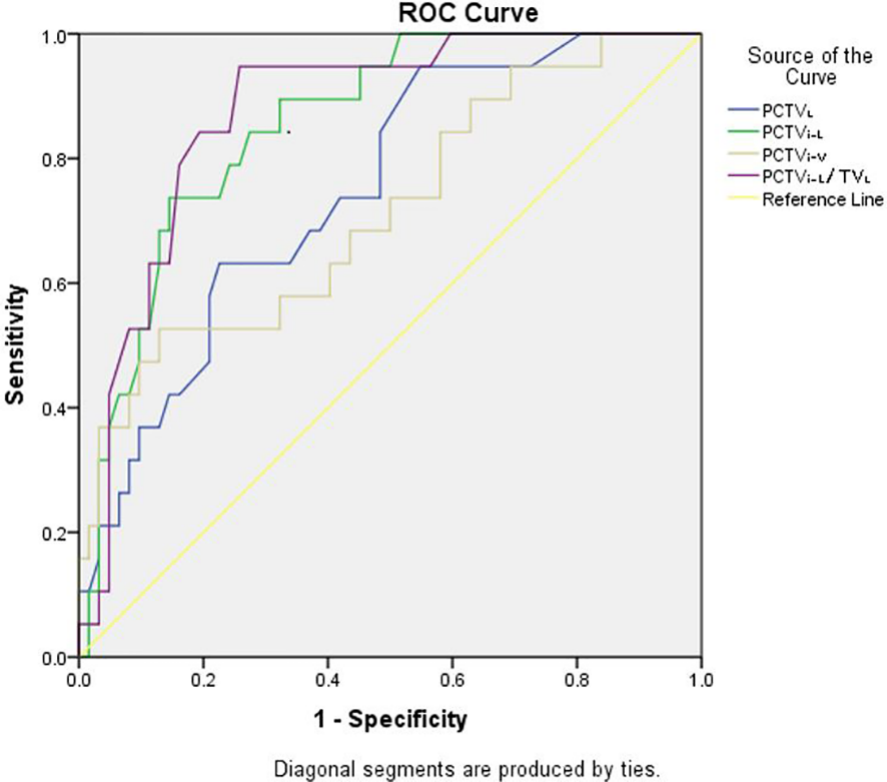
ROC analysis curves of JT IMRT for PCTV_L_, PCTV_i-V_, PCTV_i-L_ and PCTV_i-L_/TL_L_.

ROC analysis was performed for the three plans, and the details are shown in **Table 3**. The results showed that in JT IMRT, PCTV_L_ (AUC, 0.75; 95% CI, 0.63–0.87, p = 0.00), PCTV_i-V_ (AUC, 0.72; 95% CI, 0.58–0.85, p = 0.01), PCTV_i-L_ (AUC, 0.86; 95% CI, 0.77–0.94, p = 0.00) and PCTV_i-L_/TL_L_ (AUC, 0.87; 95% CI, 0.79–0.96, p = 0.00) had a power to predict the suitability of plans according to our department protocol. The details are shown in **Figure 2**.

**Table 3:** Results of receiver operating curve (ROC) analysis.

	JT IMRT		F-VMAT		P-VMAT	
	AUC	p-value	AUC	p-value	AUC	p-value
PCTV_W_	0.41	0.23	0.42	0.22	0.31	0.03
PCTV_L_	0.75	0.00	0.78	0.00	0.80	0.00
PCTV_V_	0.58	0.28	0.58	0.23	0.60	0.22
TL_V_	0.50	0.95	0.39	0.09	0.56	0.45
TL_L_	0.50	0.99	0.42	0.25	0.49	0.89
PCTV_V_/TL_V_	0.54	0.59	0.63	0.04	0.47	0.08
PCTV_i-V_	0.72	0.01	0.68	0.01	0.78	0.00
PCTV_i-L_	0.86	0.00	0.82	0.00	0.90	0.00
PCTV_i-V_/TL_V_	0.69	0.01	0.76	0.00	0.68	0.04
PCTV_i-L_/TL_L_	0.87	0.00	0.91	0.00	0.92	0.00

**Figure 3:**
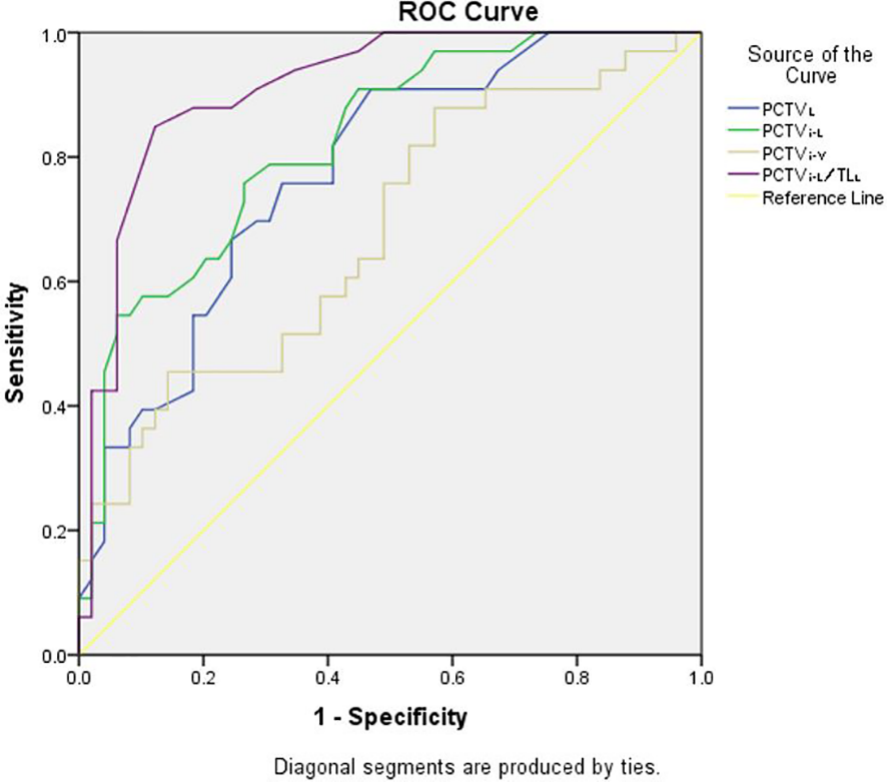
ROC analysis curves of F-VMAT for PCTV_L_, PCTV_i-V_, PCTV_i-L_ and PCTV_i-L_/TL_L_.

In F-VMAT, PCTV_L_ (AUC, 0.75; 95% CI, 0.63–0.87, p = 0.00), PCTV_i-V_ (AUC, 0.72; 95% CI, 0.58–0.85, p = 0.01), PCTV_i-L_ (AUC, 0.90; 95% CI, 0.82–0.99, p = 0.00) and PCTV_i-L_/TL_L_ (AUC, 0.92; 95% CI, 0.86–0.98, p = 0.00) had the ability to predict whether the plan meets the constraints. The details are shown in **Figure 3**.

**Figure 4:**
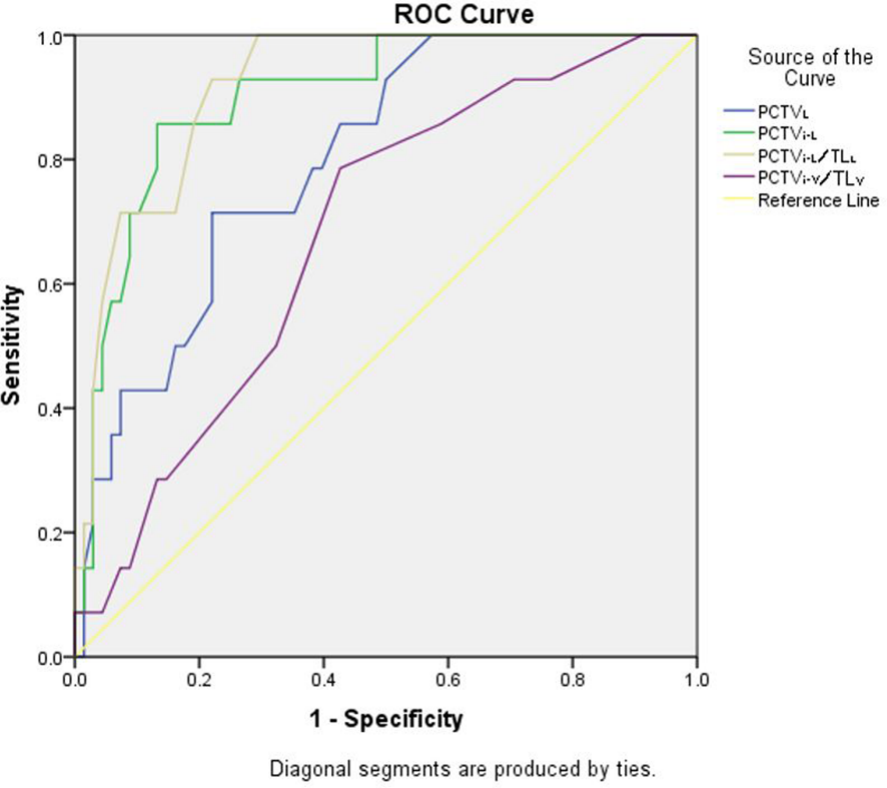
ROC analysis curves of P-VMAT for PCTV_L_, PCTV_i-V_, PCTV_i-L_ and PCTV_i-L_/TL_L_.

As shown in **Figure 4**, in P-VMAT, PCTV_L_ (AUC, 0.78; 95% CI, 0.68–0.88, p = 0.00), PCTV_i-L_ (AUC, 0.82; 95% CI, 0.74–0.91, p = 0.00), PCTV_i-V_/TL_V_ (AUC, 0.76; 95% CI, 0.66–0.86, p = 0.00), and PCTV_i-L_/TL_L_ (AUC, 0.91; 95% CI, 0.85–0.98; p = 0.00) had the ability to predict whether the plan meets the constraints.

The predictors of JT IMRT, F-VMAT, and P-VMAT were analyzed, and two predictors with the largest AUC values were finally selected: PCTV_i-L_ and PCTV_i-L_/TL_L_. After analysis, the cutoff points were determined. For JT IMRT, F-VMAT, and P-VMAT, the cutoff points of PCTV_i-L_ were 16.6, 16.75, and 15.15 cm, respectively. The cutoff points of PCTV_i-L_/TL_L_ were 0.59, 0.62, and 0.59 cm. The sensitivity, specificity, and other parameters of the cutoff points are shown in **Table 4**.

**Table 4:** The sensitivity, specificity, positive and negative predictive value, positive and negative likelihood ratios, and accuracy of the cutoff points of JT IMRT, F-VMAT, and P-VMAT.

	JT IMRT		F-VMAT		P-VMAT	
	PCTV_i-L_ (<16.6, ≥16.6)	PCTV_i-L_/TL_L_ (<0.59, ≥0.59)	PCTV_i-L_ (<16.75, ≥16.75)	PCTVi-L/TL_L_ (<0.62, ≥0.62)	PCTV_i-L_ (<15.15, ≥15.15)	PCTV_i-L_/TL_L_ (<0.59, ≥0.59)
Sensitivity (%)	74	95	76	85	86	93
Specificity (%)	85	74	73	88	87	78
Positive predictive value (%)	63	95	91	85	71	86
Negative predictive value (%)	76	75	53	88	78	81
Positive likelihood ratio	3.49	25.41	5.47	7.91	5.7	13.68
Negative likelihood ratio	1.57	2.08	2.07	5.08	1.55	1.86
Accuracy (%)	73	79	68	87	77	82

**Tables 5** and **6** show the effect of the cutoff points on the dosimetric parameters of OARs in JT IMRT, F-VMAT, and P-VMAT. In **Table 5**, it can be found that for JT IMRT, the MLD, V5, V10, V13, V20, and V30 of the total lung and MHD, V30, and V40 of the heart in the group with PCTV_i-L_ <16.6 were lower than those in the group with PCTV_i-L_ ≥16.6, and there was statistical significance. The Dmax of the spinal cord was equivalent in both groups, which had no statistical significance. For F-VMAT, the MLD, V5, V10, V13, V20, and V30 of the total lung and MHD, V30, and V40 of the heart in the group with PCTV_i-L_ <16.75 were lower than those in the group with PCTV_i-L_ ≥16.75, and there was statistical significance except for V30 of the total lung. The Dmax of the spinal cord was also equivalent in both groups. For P-VMAT, similar to JT IMRT, the MLD, V5, V10, V13, V20, and V30 of the total lung and MHD, V30, and V40 of the heart in the group with PCTV_i-L_ <15.15 were significantly lower than those in the group with PCTV_i-L_ ≥15.15. The Dmax of the spinal cord was comparable in both groups.

**Table 5:** Dosimetric parameters with grouping PCTV_inferior_ length.

	JT IMRT			F-VMAT			P-VMAT		
	PCTV_i-L_ <16.6	PCTV_i-L_ ≥16.6	p-value	PCTV_i-L_ <16.75	PCTV_i-L_ ≥16.75	p-value	PCTV_i-L_ <15.15	PCTV_i-L_ ≥15.15	p-value
MLD (Gy)	8.81 ± 1.40	11.01 ± 2.09	**<0.001**	8.64 ± 1.55	10.52 ± 2.23	**<0.001**	8.16 ± 1.14	10.49 ± 1.83	**<0.001**
Total lung V_5_ (%)	38.74 ± 7.55	51.16 ± 11.53	**<0.001**	40.02 ± 9.74	55.53 ± 15.54	**<0.001**	34.24 ± 6.15	50.37 ± 11.85	**<0.001**
Total lung V_10_ (%)	27.88 ± 5.59	38.08 ± 9.58	**<0.001**	29.09 ± 6.24	39.25 ± 12.38	**<0.001**	24.78 ± 3.61	34.56 ± 8.48	**<0.001**
Total lung V_13_ (%)	24.61 ± 4.42	33.12 ± 8.40	**<0.001**	24.74 ± 4.53	31.26 ± 8.44	**<0.001**	21.56 ± 3.05	29.03 ± 6.27	**<0.001**
Total lung V_15_ (%)	22.32 ± 3.91	30.00 ± 7.43	**<0.001**	22.14 ± 3.82	26.77 ± 6.29	**<0.001**	19.67 ± 2.81	25.74 ± 5.25	**<0.001**
Total lung V_20_ (%)	17.24 ± 2.62	21.26 ± 4.67	**<0.001**	16.28 ± 2.90	17.92 ± 3.46	**0.02**	15.47 ± 2.35	18.67 ± 3.54	**<0.001**
Total lung V_30_ (%)	8.55 ± 2.04	9.80 ± 2.20	**0.01**	7.35 ± 1.92	7.39 ± 1.96	0.73	8.52 ± 1.75	9.72 ± 2.73	**0.04**
Spinal Cord D_max_ (Gy)	44.07 ± 2.30	44.04 ± 2.98	0.68	42.17 ± 2.82	41.42 ± 4.63	0.53	44.17 ± 2.14	43.20 ± 2.79	0.11
Heart MHD (Gy)	4.39 ± 3.09	12.01 ± 6.18	**<0.001**	3.89 ± 2.76	8.50 ± 4.57	**<0.001**	4.87 ± 3.40	12.79 ± 5.88	**<0.001**
Heart V_30_ (%)	5.11 ± 5.37	16.59 ± 9.60	**<0.001**	3.47 ± 3.91	8.30 ± 4.89	**<0.001**	5.85 ± 5.94	17.03 ± 8.30	**<0.001**
Heart V_40_ (%)	2.62 ± 2.92	7.74 ± 4.57	**<0.001**	1.71 ± 2.12	4.09 ± 2.54	**<0.001**	3.12 ± 3.52	8.69 ± 4.17	**<0.001**

Bold value means p < 0.05 and it was considered statistically significant.

**Table 6:** Dosimetric parameters with grouping PCTV_inferior_ length/total lung length.

	JT IMRT			F-VMAT			P-VMAT		
	PCTV_i-L_/TL_L_ <0.59	PCTV_i-L_/TL_L_ ≥0.59	p-value	PCTV_i-L_/TL_L_ <0.62	PCTV_i-L_/TL_L_ ≥0.62	p-value	PCTV_i-L_/TL_L_ <0.59	PCTV_i-L_/TL_L_ ≥0.59	p-value
MLD (Gy)	8.48 ± 1.28	10.83 ± 1.80	**<0.001**	8.35 ± 1.32	11.14 ± 1.92	**<0.001**	8.14 ± 1.25	9.21 ± 2.01	**<0.001**
Total lung V_5_ (%)	36.39 ± 6.20	50.82 ± 9.49	**<0.001**	38.18 ± 7.96	59.96 ± 12.90	**<0.001**	33.60 ± 5.79	41.64 ± 12.03	**<0.001**
Total lung V_10_ (%)	26.39 ± 4.65	37.17 ± 8.40	**<0.001**	27.82 ± 4.85	42.24 ± 11.30	**<0.001**	24.75 ± 4.05	29.02 ± 8.00	**<0.001**
Total lung V_13_ (%)	23.52 ± 3.85	32.15 ± 7.36	**<0.001**	23.86 ± 3.74	33.26 ± 7.73	**<0.001**	21.60 ± 3.50	24.82 ± 6.19	**<0.001**
Total lung V_15_ (%)	21.58 ± 3.35	28.78 ± 6.93	**<0.001**	21.46 ± 3.27	28.27 ± 5.78	**<0.001**	19.72 ± 3.24	22.36 ± 5.24	**<0.001**
Total lung V_20_ (%)	16.84 ± 2.61	20.66 ± 4.11	**<0.001**	15.93 ± 2.59	18.61 ± 3.49	**<0.001**	15.57 ± 2.65	16.99 ± 3.56	**<0.001**
Total lung V_30_ (%)	8.50 ± 2.12	9.50 ± 2.08	**0.04**	7.18 ± 1.83	7.63 ± 2.05	0.20	8.72 ± 2.05	9.13 ± 2.46	0.52
Spinal cord D_max_ (Gy)	44.13 ± 2.46	43.97 ± 2.59	0.88	42.21 ± 3.15	41.27 ± 4.48	0.39	44.12 ± 2.17	42.99 ± 6.18	0.31
Heart MHD (Gy)	3.64 ± 2.48	10.84 ± 5.73	**<0.001**	3.67 ± 2.41	9.35 ± 4.33	**<0.001**	4.43 ± 3.00	8.77 ± 5.55	**<0.001**
Heart V_30_ (%)	3.84 ± 4.24	15.01 ± 9.00	**<0.001**	3.15 ± 3.55	9.32 ± 4.51	**<0.001**	5.06 ± 5.17	11.67 ± 8.26	**<0.001**
Heart V_40_ (%)	2.01 ± 2.56	7.09 ± 4.24	**<0.001**	1.64 ± 2.16	4.48 ± 2.26	**<0.001**	2.75 ± 3.30	6.04 ± 4.44	**<0.001**

Bold value means p < 0.05 and it was considered statistically significant.

The analysis results of PCTV_i-L_/TL_L_ are shown in **Table 6**. For JT IMRT, F-VMAT, and P-VMAT, the cutoff points were different. Nevertheless, in the group with lower PCTV_i-L_/TL_L_, MLD, V5, V10, V13, V20, and V30 of the total lung and MHD, V30, and V40 of the heart were all lower. Except for V30 of total lung in F-VMAT and P-VMAT, there was statistical significance.

In summary, when PCTV_i-L_ <15.15, JT IMRT, F-VMAT, and P-VMAT can be used for plan design. According to the results of this research, we recommend using P-VMAT because the lung dose of this technology is generally lower than that of the other two technologies on the premise of equal target coverage. When 15.15 ≤ PCTV_i-L_ <16.6, JT IMRT and F-VMAT can be used. When 16.6 ≤ PCTV_i-L_ <16.75, F-VMAT seems to be more suitable for plan design. When PCTV_i-L_ >16.75, since the cutoff value of F-VMAT is the largest, planners can adopt F-VMAT first. If the plan does not meet the requirements, they can try to use fixed-jaw intensity modulated radiotherapy or other non-photonic technology, such as proton and heavy ion technology. For PCTV_i-L_/TL_L_, when PCTV_i-L_/TL_L_ <0.59, JT IMRT, F-VMAT, and P-VMAT can be used for plan design; we also recommend using P-VMAT. When 0.59 ≤ PCTV_i-L_/TL_L_ <0.62, F-VMAT technology can be adopted for planning design. For planning, when the radiotherapy techniques recommended by PCTV_i-L_ and PCTV_i-L_/TL_L_ are inconsistent, it is necessary to rely on the physicist. Physicists need to combine their own clinical experience, make careful judgments, discuss in detail, and finally determine which technology is more suitable for the plan.

## Discussion

In this study, we evaluated the effect of PCTV_W_, PCTV_L_, PCTV_V_, TL_V_, TL_L_, PCTV_V_/TL_V_, PCTV_i-V_, PCTV_i-L_, PCTV_i-V_/TL_V_, and PCTV_i-L_/TL_L_ on total lung dose among T-shaped UMEC in JT IMRT, F-VMAT, and P-VMAT. The goal was to investigate whether there were one or more predictors that can be used to predict whether the plans would be able to meet dose constraints and find the cutoff points of the predictors. The results showed that for JT IMRT, F-VMAT, and P-VMAT, PCTV_i-L_ and PCTV_i-L_/TL_L_ were the two predictors with the highest predictive ability. Among JT IMRT, F-VMAT, and P-VMAT, the cutoff points of PCTV_i-L_ were 16.6, 16.75, and 15.15, respectively. The cutoff points of PCTV_i-L_/TL_L_ were 0.59, 0.62, and 0.59, respectively. The results of the study can be used to provide a reference for T-shaped UMEC patients to choose technology under the premise of meeting the lung dose constraints.

Radiotherapy for EC is associated with the risk of radiation pneumonitis. Studies have shown that radiation pneumonia is related to dosimetric parameters such as MLD, V5, V20, and V30 of the total lung (25–27). Shally et al. (25) found that MLD and V20 of the total lung were predictors of RP. Wang et al. (26) reported that V5, V20, and MLD of the total lung were highly correlated with RP. Therefore, in the radiotherapy of EC, it is essential to obtain a lung dose that meets the constraints, which can effectively reduce the incidence of RP. The two predictors PCTV_i-L_ and PCTV_i-L_/TL_L_ found in our research can effectively predict whether the treatment plan can meet the constraints of total lung. This may have certain potential clinical significance in the planning process.

The results in **Table 2** show that for T-shaped UMEC patients, P-VMAT could significantly reduce total lung dose when target coverage was comparable. This result is consistent with our previous research (20, 28). Choi et al. (29) found that compared with IMRT, VMAT can reduce V20 and V30 of the total lung but will increase V5 and V10 of the total lung. Our research also proved this result.

Gong et al. (30) studied the feasibility of using the deep breath-hold technique to reduce lung and cardiac dose in the treatment of EC with VMAT. It was found that the deep breath-holding technique increased the patient’s lung volume and significantly reduced V10, V20, V30, V40, and MLD of total lung compared with free breathing. Bolukbas et al. (22) found that lung volume and lung/PTV can predict whether the TOMO plan meets QUANTEC dose constraints. In this study, the predictive ability of lung volume and lung/PTV was analyzed. The results showed that the predictive ability of these two factors was low in JT IMRT, F-VMAT, and P-VMAT. The reason may be that the patients enrolled in this study were patients with T-shaped UMEC. Bolukbas et al. (22) studied upper EC and only delineated the virtual radiotherapy target, excluding lymph node metastasis.

In our study, the evaluation factors of total lung considered in our department were V5, V20, and MLD. According to the results shown in **Tables 5** and **6**, for the three technologies, the group with lower PCTV_i-L_ and PCTV_i-L_/TL_L_ could significantly reduce MLD, V5, V10, V13, V15, V20, and V30 of the total lung. This result was similar to that of Bolukbas et al. (22). Gong et al. (30) found that the lung volume increased, but the heart dose did not decrease. Bolukbas et al. (22) found that for patients with upper EC, heart dose (Dmean, V5, V20, V30, and V45) can be significantly reduced in the group with higher lung/PTV and lung volume value. In our study, the results showed that patients with lower PCTV_i-L_ and PCTV_i-L_/TL_L_ had significantly lower MHD, V30, and V40 of heart. According to these results, it should be considered that the group with lower PCTV_i-L_ and PCTV_i-L_/TL_L_ not only had a lower total lung dose but also a better protected heart.

There are some limitations in this study. The enrolled patients are all patients with T-shaped UMEC. The cutoff points of our research may not be applicable to distal tumors. This study is mainly based on the anatomical parameters of target to find factors that can predict lung dose. If more factors in the treatment planning process were considered, the results may be more universal. The three plans of JT IMRT, F-VMAT, and P-VMAT in the study were designed by our dosimetrists based on years of clinical experience. The cutoff points in this study were based on the results of these plans. For centers that adapt different methods to design plans, the cutoff points may vary. However, the method used in this study is universal, and different centers can determine the cutoff points according to the actual situation of their centers.

## Conclusion

This article investigates the existence of predictors to achieve acceptable lung dose in patients with T-shaped UMEC, using JT IMRT, F-VMAT, and P-VMAT. PCTV_i-L_ <16.6 and PCTV_i-L_/TL_L_ <0.59, PCTV_i-L_ <16.75 and PCTV_i-L_/TL_L_ <0.62, and PCTV_i-L_ <15.15 and PCTV_i-L_/TL_L_ <0.59 can respectively predict whether JT IMRT, F-VMAT, and P-VMAT plans would meet the total lung dose constraints of our department protocol in patients with T-shaped UMEC. The results could provide data reference for our department to use different techniques to treat patients with T-shaped UMEC. At the same time, the method used in this article can also be transferred to lower esophageal cancer and other radiotherapy departments.

## Data Availability Statement

The original contributions presented in the study are included in the article. Further inquiries can be directed to the corresponding authors.

## Author Contributions

YS: data collection, statistical analysis, and writing and revising the manuscript. HC, AF, HG, HW, YD, and YH: patient administration and critical revision of the manuscript. QK and ZX: study design, critical revision of the manuscript, and funds collection. All authors contributed to the article and approved the submitted version.

## Conflict of Interest

The authors declare that the research was conducted in the absence of any commercial or financial relationships that could be construed as a potential conflict of interest.

## Publisher’s Note

All claims expressed in this article are solely those of the authors and do not necessarily represent those of their affiliated organizations, or those of the publisher, the editors and the reviewers. Any product that may be evaluated in this article, or claim that may be made by its manufacturer, is not guaranteed or endorsed by the publisher.
